# The effectiveness of social-themed picture book reading in promoting children’s prosocial behavior

**DOI:** 10.3389/fpsyg.2025.1569925

**Published:** 2025-04-14

**Authors:** Honglin Chen, Dannuo Lyu, Liqi Zhu

**Affiliations:** ^1^State Key Laboratory of Cognitive Science and Mental Health, Institute of Psychology, Chinese Academy of Sciences, Beijing, China; ^2^Department of Psychology, University of Chinese Academy of Sciences, Beijing, China

**Keywords:** shared reading, prosocial behavior, empathy, preschool children, picture books

## Abstract

**Introduction:**

There is a need for an effective and low-cost approach to promote prosocial behavior in preschool children. This study examines the effectiveness of parent-child shared reading of socially themed picture books on prosocial behavior in preschoolers, and explores the mediating role of empathy.

**Methods:**

Sixty children (aged 4-5 years) and their parents were randomly assigned to either the intervention group, which read socially themed picture books, or the control group, which read books on other topics. Shared reading sessions took place twice a week for eight weeks. Prosocial behavior tasks and the Empathy Questionnaire (EmQue) were administered pre- and post-intervention.

**Results:**

Children in the intervention group scored significantly higher on prosocial behavior and empathy than those in the control group. Mediation analysis further revealed that empathy fully mediated the relationship between shared reading of socially themed picture books and prosocial behavior.

**Discussion:**

These findings highlight the role of empathy as a key mechanism through which socially themed picture books promote prosocial behavior. This research provides valuable insights for family education, highlighting a low-cost approach that promotes children’s social development through everyday storytelling without the need for specialized training.

## Introduction

Prosocial behaviors are typically defined as voluntary actions intended to benefit others, such as helping, comforting, and sharing (Eisenberg, 2006). Although early childhood is a crucial period for the development of these behaviors (Hay et al., 2004), existing interventions for prosocial behavior primarily focus on adolescents (e.g., Shin and Lee, 2021; Laguna et al., 2023). Research has indicated a strong relationship between prosocial behavior and several aspects of adaptive development, including social acceptance and friendship (Clark and Ladd, 2000; Sebanc, 2003), psychosocial adjustment (Hay and Pawlby, 2003), and academic achievement (Caprara et al., 2000). Additionally, previous research has affirmed that children exhibiting concern and compassion are more likely to grow into compassionate adults (Denham, 1986). Although this association highlights the importance of fostering prosocial skills in children from a young age, most studies in this area either focus on clinical populations (e.g., Crozier and Tincani, 2007; Eden and Oren, 2021), or are part of large-scale programs where prosocial behavior is merely one of the outcome measures (e.g., Flook et al., 2015; Schell et al., 2015; Kim et al., 2020). Additionally, many interventions require interventionists with specialized training (e.g., Leung et al., 2019) or specific settings (e.g., Ornaghi et al., 2015). Therefore, there remains a need to develop an effective approach that can be easily implemented in daily educational settings without requiring professionally trained interventionists and that specifically targets prosocial behavior in non-clinical preschool children.

One effective way to promote prosocial behavior during early childhood may be through reading socially themed picture books. Several experimental studies have validated that reading socially themed picture books has a significant positive impact on the development of prosocial behavior and altruistic giving in preschoolers (Kohm et al., 2016; Larsen et al., 2018). Furthermore, a study has affirmed that the emotional explanations of mothers during parent–child shared reading are positive predictors of prosocial behavior in children during play, whereas emotional comments by mothers during reading negatively correlate with the physical aggression of children (Garner et al., 2008). Socially themed picture books provide more opportunities for parent–child dyads to discuss and comment on emotions, the effect of which may be mediated by increasing the empathy of children.

The present research tested the effectiveness and mechanisms of an intervention based on parent–child shared reading of socially themed picture books to promote prosocial behavior of children.

### Picture book reading and prosocial behavior

Stories are a powerful tool for socialization, shaping and transmitting moral values across generations (Yao and Enright, 2020). Children’s picture books cover a wide range of themes; however, not all themes may contribute to promoting prosocial behavior in children. The theme of a picture book refers to its central idea or purpose and the major concepts it seeks to convey. Common themes include daily routines, social issues, popular science, and cognitive or creative topics. In particular, socially themed books focus on stories addressing aspects of social life and interpersonal communication, assisting young children explore and comprehend common social interactions through storytelling. These stories typically encompass interpersonal behaviors and the emotional context underlying those behaviors.

Reading picture books with social content has been found to promote prosocial behavior in children. Larsen et al. (2018) found that when experimenters read prosocial storybooks with human characters to children, the children were more likely to share stickers in a follow-up sharing task. Similarly, whether children heard a story in which the main character was rewarded for sharing or one in which the main character faced negative consequences for refusing to share, they were more willing to share candy in the sharing task compared to the control group (Yao and Enright, 2020). Study also found that moral stories featuring internal negative reinforcement, where the main character feels guilty for not sharing, had the strongest effect on promoting children’s sharing behavior (Yu et al., 2024). Previous studies have also found that social-themed picture books are also closely linked to children’s helping behavior. Reading moral stories with a helping theme increased children’s willingness to help (Du and Hao, 2018). Additionally, Kohm et al. (2016) confirmed that children exhibited more helping behaviors and engaged in higher-quality social interactions after being read storybooks with moral lessons and prosocial content. In these short-term experiments, a single reading of picture books significantly increased the children’s subsequent levels of prosocial behavior. Despite the lack of exploration into the underlying mechanisms, the above findings implicitly suggest the possibility of intervening in the development of prosocial behavior in young children through the reading of socially themed picture books.

In this study, we focus on parent–child shared reading as the primary way children engage with social-themed picture books, as it is a cultural routine involving adults and young children worldwide. Research has shown that this interactive parent–child reading format plays a critical role in facilitating children’s social–emotional development (Curenton and Craig, 2011). The frequency of parent–child shared reading significantly predicts higher levels of social functioning (Farver et al., 2006) and theory of mind scores (Lenhart and Richter, 2024). During parent–child shared reading, discussions about social–emotional issues positively predict children’s social understanding and prosocial behavior (Aram et al., 2017). Conversely, children whose parents lack involvement at home, including a low frequency of shared reading, tend to exhibit higher levels of problem behaviors (Fleming et al., 2002). Video-coded observations of parent–child shared reading of social-themed picture books have revealed that most mothers discussed the emotional consequences of wrongdoing, addressing both interpersonal and material moral domains, suggesting that shared reading of social-themed picture books serves as a key avenue for mother–child moral communication (Gomes et al., 2022).

In summary, the current study sought to examine the effectiveness of an intervention involving parent–child shared reading of socially themed picture books on prosocial behavior in preschool children. We hypothesized that children in the intervention group would exhibit a significantly greater increase in prosocial behavior after the intervention than children in the control group who read picture books with other themes.

### The mediating role of empathy

Empathy is the ability to understand and share the feelings of others and to respond appropriately to their situations (Hoffman, 2000). It is a complex construct that encompasses multiple components. Most researchers agree that empathy includes both cognitive empathy, which encompasses recognizing and understanding the emotions of other individuals, and emotional empathy, which refers to experiencing the emotions of others (Decety and Meyer, 2008). Some researchers have also affirmed that empathy is closely linked to behavioral responses and should be divided into three dimensions, namely, cognitive, emotional, and behavioral empathy (Zhang et al., 2014).

The simulation of social experience theory suggests that simulated experiences generated through literary reading can enhance the empathic experiences of readers, thereby fostering the development of social cognitive abilities (Mar, 2011; Oatley, 2016). For preschool children, picture books are a primary reading material, effectively conveying rich emotions through vivid illustrations and concise text. Immersing in the story context of picture books, children can simulate experiences, which enhances their empathic experiences. Compared with picture books with other themes, socially themed picture books comprise more interpersonal behaviors and the emotional context underlying these behaviors, which can evoke stronger emotional responses. Studies have affirmed that narratives with emotional content can evoke empathy in readers and promote a sense of immersion in reading, thereby activating emotional empathy-related networks, including the anterior insula, medial prefrontal cortex, amygdala, secondary somatosensory cortex, and inferior frontal gyrus (Hsu et al., 2014).

Socially themed picture books frequently depict the actions, emotions, and psychological states of others, offering parents opportunities to help children understand the causal relationship between emotions and behaviors during storytelling. On the one hand, the act of parent–child reading together increases the frequency of parent–child interactions, which is linked to the development of emotional empathy (Decety and Holvoet, 2021). On the other hand, parents tend to discuss the emotions of others more frequently during shared reading than in everyday conversations (Sabbagh and Callanan, 1998). Conversations about emotional states during storytelling, especially those enriched with emotional lexicon, can enhance the ability of children to understand various aspects of emotions (Grazzani and Ornaghi, 2011), which is crucial for developing cognitive empathy in children (Decety and Holvoet, 2021).

Empathy is vital for the development of prosocial behavior. The Eisenberg proximal social behavior developmental model explains how individuals decide whether to engage in prosocial behaviors through a series of psychological processes, emphasizing that people with high levels of empathy are more attuned to the emotional states of others, which motivates them to help (Eisenberg and Miller, 1987). Numerous studies have confirmed that empathy is associated with prosocial behavior. For instance, Malti et al. (2009) affirmed that empathy measured by various methods (self-reports, teacher reports, and parent reports) was significantly positively correlated with prosocial behavior measured by the same methods. Denham (1986) employed naturalistic observations and individual interviews to demonstrate that emotional cognition can still significantly and independently predict prosocial behavior in 2- to 3-year-old children, even when controlling for gender and grade differences. Current research in affective neuroscience validates that integrating compassion interventions with both affective and cognitive empathy provides the most effective approach to fostering prosocial behavior in individuals (Stevens and Taber, 2021).

Empathy may mediate the relationship between shared reading of socially themed picture books and prosocial behavior. As previously mentioned, this form of shared reading increases the frequency with which parents discuss emotions with their children, thereby fostering the development of children’s empathy—a trait closely linked to prosocial behavior. For instance, a similar study has validated that toddlers whose parents frequently prompt them to reflect on and discuss the emotions depicted in picture books are quicker and more frequent in their helping and sharing behaviors with a needy adult (Brownell et al., 2013).

Based on these insights, we hypothesized that empathy plays a mediating role in the process through which reading socially themed picture books promotes the development of prosocial behavior in children.

### Present study

Previous studies have found that reading socially themed picture books can enhance prosocial behavior of children. Nonetheless, there is a lack of intervention studies confirming their long-term effects, and their underlying mechanisms have not been sufficiently explored. We hypothesize that when parents engage in shared reading of socially themed picture books with their children, the children are exposed to rich emotional content and engage in discussions about the emotional states of characters, which is associated with improved empathy. In turn, this empathy further promotes prosocial behavior. Thus, the present research aims to enhance the prosocial behavior of preschool children through parent–child shared reading of socially themed picture books and examine the mediating role of empathy in this process. The following hypotheses are proposed:

*H1*: Children in the intervention group, who read socially themed picture books with their parents, will exhibit a significantly greater increase in prosocial behavior after the intervention compared with children in the control group, who read picture books exploring other themes.

*H2*: Empathy plays a mediating role between parent–child shared reading of socially themed picture books and the prosocial development of children.

## Method

### Participants

We conducted power analyses using G*Power (Faul et al., 2007) to estimate the expected effects of parent–child shared reading of socially themed picture books on prosocial behaviors. The results indicate that given an alpha of 0.05 and a power of 0.95, a total sample of 54 participants will be required to detect an effect size of *η*^2^ = 0.44 when testing the differences between the intervention and control groups, as reported in a similar study (Li et al., 2023).

In a kindergarten located in a southwestern city of China, we recruited 60 middle school children, comprising 32 boys and 28 girls, with an average age of 55.27 ± 3.81 months. Of the participants, 58% were only children, and 85% of the primary caregivers were their parents. The educational qualifications of the parents were relatively high, with 86.6% having completed a bachelor’s degree or higher. Parent–child dyads were assigned at random to the intervention and control groups, with 30 dyads in each group. No significant differences were found in demographic variables, empathy, or prosocial behavior between the intervention and control groups at the pretest (Table 1).

**Table 1:** Descriptive statistics for all variables by group condition at pretest.

		Intervention group (*n* = 30)	Control group (*n* = 30)	*χ^2^/t*	*p*
Child age in months (SD)		55.80 (3.67)	54.73 (3.92)	−1.08	0.15
Gender	Male %	43.33%	53.33%	0.60	0.44
	Female %	56.67%	46.67%		
Only child or not	Yes %	56.67%	66.67%	0.64	0.43
	No %	43.33%	33.33%		
Primary caregiver ratio	Parents %	86.67%	83.33%	0.13	0.72
	Others %	13.33%	16.67%		
Maternal education	Bachelor’s degree or above %	80.00%	73.33%	0.37	0.54
	Below bachelor’s degree %	20.00%	26.67%		
EmQue score (SD)		59.03 (8.02)	56.82 (6.53)	−0.33	0.10
Prosocial behavior score (SD)		2.00 (1.01)	2.47 (1.50)	1.41	0.05
Frequency of reading per week (SD)		3.87 (1.20)	3.80 (1.38)	0.24	0.41
Duration of reading per session (min)		26.67 (7.11)	25.73 (12.08)	−0.597	0.28

The recruitment period for this study begins on March 4, 2024 and ends on May 30, 2024. Furthermore, the researchers distributed invitations for the picture book shared reading study, with a consent form included on the first page. All parents provided written informed consent prior to the commencement of the study, which was conducted in accordance with the Declaration of Helsinki.

### Materials

#### Empathy

##### Empathy questionnaire (EmQue)

We employed the EmQue to measure the empathy abilities of children (Rieffe et al., 2010). This questionnaire was specifically designed for young children and is based on the empathy theories (Hoffman, 2000; De Waal, 2008). It encompasses three dimensions: cognitive, emotional, and behavioral empathy. Chinese scholar translated and adapted the questionnaire for Chinese preschool children and subsequently conducted reliability and validity tests (Yan et al., 2019). The results demonstrated that the revised Chinese version of the EmQue for preschoolers had good reliability and validity, making it a suitable tool for measuring empathy in Chinese preschool children. The questionnaire comprises 20 items regarding children’s interactions with their peers. Using a 5-point Likert scale (1 = never, 5 = always), parents rated how closely each situation described in the questionnaire aligned with their children’s experiences. The sum of all items was calculated as an overall measure of empathy, with high scores indicating better empathy abilities. In the current study, the internal consistency coefficient of the total score was good (Cronbach’s *α* = 0.795).

#### Prosocial behavior

##### Helping task

Based on the approach employed by Reschke et al. (2023), a child was seated opposite the experimenter, who adhered to a scripted series of actions without offering encouragement or praise during the task. The experimenter “accidentally” knocked a pen holder off the table, causing the pens to scatter on the floor, and then nervously exclaimed, “Oops, my pens fell!” If the child did not offer assistance within 5 s, the experimenter prompted, “Could you help me pick them up?” The experimenter then observed the child’s behavior and scored it based on the following criteria: 2 = the child actively picked up the pens before the experimenter asked for help, 1 = the child picked up the pens after the examiner asked for help, and 0 = the child did not help at all.

##### Sharing task

Adapted from the dictator game employed by Benenson et al. (2007), the children were seated in front of the experimenter, who presented a collection of stickers and informed them that they could choose their seven favorite stickers as a reward for participating in the game. After they had selected the stickers, the experimenter explained that they would be visiting another kindergarten the next day to play with other children; however, there were not enough stickers to go around. Hence, the children were told they could keep all seven stickers or share some with the children at the other kindergarten. Subsequently, the experimenter provided the children with two envelopes, one with a smiley face, and instructed them to place the stickers they wished to keep in the envelope with the smiley face and any stickers they wanted to share (if any) in the envelope without the smiley face. The experimenter emphasized the anonymity of the children’s decisions and then turned away until the children informed they had finished distributing the stickers. Finally, the experimenter collected the envelope without the smiley face and opened it after the experiment concluded to record the number of stickers shared by them.

The order of the helping and sharing tasks was counterbalanced across subjects. The scores from both tasks were then combined to calculate the total prosocial behavior score.

#### Intervention materials

Thirty-two picture books were selected by PhD and master’s degree holders with extensive experience in the field of social development of children. All books were chosen from the list of picture books recommended by the Ministry of Education of the People’s Republic of China for children aged 3–6 (Ministry of Education of the People’s Republic of China, 2021). Sixteen of the socially themed picture books focused on four categories: cooperation, sharing, helping, and friendship. The remaining 16 picture books, themed around science, creativity, and daily habits, served as the control group. The books in the control group were comparable to those in the experimental group in terms of length, ratio of illustrations to text and interactive format, but did not include social–emotional content. The original text of the picture books is in Chinese, and their translated titles are listed in Table 2.

**Table 2:** Picture books list with different themes.

Picture books themes		Picture books list
Social theme	Cooperation	The Flavor of the Moon; Turn a Carrot; Ants and Watermelon
	Sharing	Rainbow-Colored Flowers; Granny Flower; Stone Soup; Grandpa Shanping’s Strawberries; Half for One is Perfect
	Helping	Badger’s Gift; Red Shoes; Can You Help Me?; You Help Me, I Help You
	Friendship	Best Friends: How to Build and Maintain Friendships; I Have a Friendship for Rent; The Red Balloon is Gone; Amo’s Sick Day
Science, creativity, and life habit themes		Mr. Water’s Fantastic Journey; Where’s Mrs. Air?; Mr. Sun in Clothes; Mr. Tree Grows Up; I’m Wearing Glasses; Point, Point, Point; Secret of the Night; Winding Busses; Birthday Moon; Olympics, Soccer, Airplane Travel, Night, Chocolate, Ski Resort, Ocean — Kiss Science Library Series

#### Procedure

Prior to the intervention, the children were assessed on their prosocial behavior using an on-site test, and their parents completed the EmQue. Each group received 16 picture books, with the intervention group receiving socially themed picture books and the control group receiving picture books exploring other themes. The parents and children in both groups were instructed to read two books together per week for 8 weeks. The duration was determined based on previous shared reading interventions that demonstrated significant effects within similar time frames (e.g., Lingwood et al., 2020; Salley et al., 2022). The order in which the books were read was not specified for either group. Each book was read once, with each session lasting 20 to 30 min.

During the intervention, the experimenter monitored the reading progress of both groups through WeChat groups. The parents recorded the reading sessions and submitted weekly reports to the group, with a final report submitted at the end of the eight-week period. The records included information on the chaperone, session duration, questions and interactions that emerged during the session, and session photos. As presented in Table 1, no significant difference was found between the intervention and control groups in terms of the frequency of picture book reading per week and duration of each session.

After the intervention ended, the experimenter followed up on the social behavior of the 60 children, and their parents completed the EmQue again.

#### Analysis

The present research employed SPSS 24.0 for data analysis, including descriptive statistics, difference test analysis, and mediation test. A repeated-measure difference analysis was used to examine the effects of shared reading of picture books with different themes on prosocial behavior of children. Additionally, a mediation model was constructed to explore the mediating role of children’s empathy in the relationship between parent–child socially themed picture book reading and children’s prosocial behavior.

## Results

### Descriptive statistics

To clearly present the prosocial behavior and empathy scores of the intervention and control groups at the pretest and posttest, we calculated the mean and standard deviation for each group (Table 3).

**Table 3:** Means and standard errors for prosocial behavior and empathy at pretest and posttest for the intervention and control groups.

Content		Group			
		Intervention group		Control group	
		Pretest	Posttest	Pretest	Posttest
Prosocial behavior	Help	1.50 (0.51)	1.66 (0.47)	1.57 (0.50)	1.67 (0.49)
	Share	0.50 (1.00)	1.04 (1.18)	0.90 (1.39)	0.93 (1.53)
	Total prosocial behavior score	2.00 (1.01)	2.70 (1.23)	2.47 (1.50)	2.60 (1.69)
Empathy	Cognitive empathy	20.60 (3.91)	22.46 (3.45)	20.56 (3.35)	20.96 (4.01)
	Emotional empathy	15.20 (4.91)	18.40 (4.97)	14.30 (3.95)	15.63 (3.25)
	Behavioral empathy	18.86 (4.21)	19.00 (3.92)	18.96 (3.75)	18.53 (3.95)
	Total empathy score	59.03 (8.64)	61.30 (8.13)	56.82 (5.94)	56.99 (6.03)

### Intervention effects on preschoolers’ prosocial behavior and empathy

To validate the effect of the intervention on preschool children’s prosocial behavior and empathy, we conducted a repeated-measures ANOVA, with time (pretest or posttest) being a within-subject factor, group condition (intervention or control group) being a between-subjects factor, and children’s gender being a covariate. Prosocial behavior and empathy scores from the pretest and posttest served as the dependent variables, respectively.

**Figure 1:**
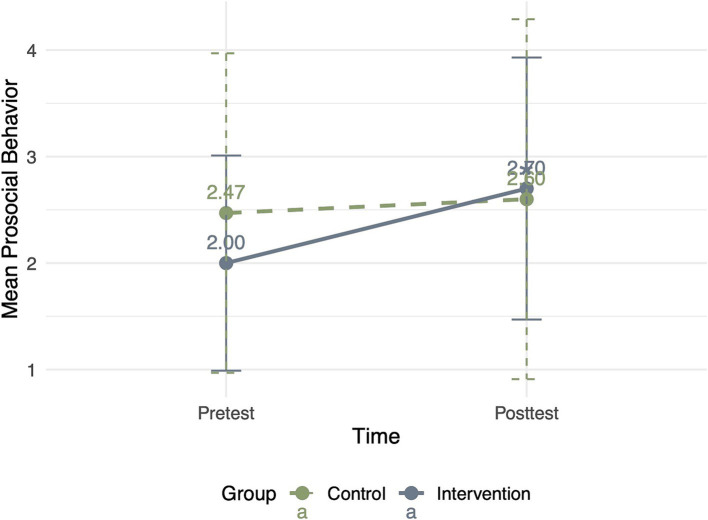
Changes in prosocial behavior from pretest to posttest in the intervention and control groups.

For prosocial behavior, the results confirmed that the main effects of group (*F* = 0.506, *p* = 0.480) and time (*F* = 0.020, *p* = 0.888) were insignificant. The interaction between the group and time was significant (*F* = 4.633, *p* = 0.036, *df* = 55, *η^2^* = 0.077). Children in the intervention group (*M*_pretest_ = 2.00 ± 1.01; *M*_posttest_ = 2.70 ± 1.23) exhibited a significant increase in prosocial behavior compared with children in the control group (*M*_pretest_ = 2.47 ± 1.50; *M*_posttest_ = 2.60 ± 1.69) (Figure 1). The results confirmed that reading socially themed picture books significantly promoted children’s prosocial behavior, supporting H1.

**Figure 2:**
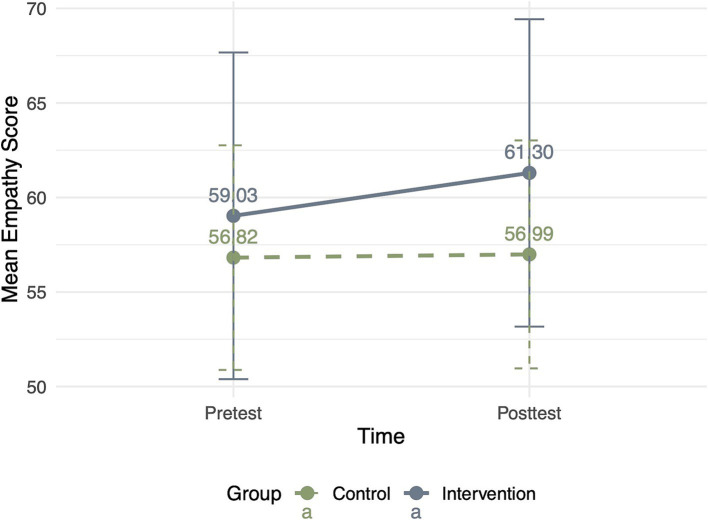
Changes in empathy from pretest to posttest in the intervention and control groups.

For empathy, the results confirmed that the main effects of group (*F* = 0.171, *p* = 0.680) and time (*F* = 0.786, *p* = 0.379) were insignificant. The interaction between the group and time was significant (*F* = 8.606, *p* = 0.005, *df* = 55, *η^2^* = 0.137). Children in the intervention group (*M*_pretest_ = 59.03 ± 8.64; *M*_posttest_ = 61.30 ± 8.13) showed a significant increase in empathy compared with children in the control group (*M*_pretest_ = 56.82 ± 5.94; *M*_posttest_ = 56.99 ± 6.03) (Figure 2). The results demonstrated that reading socially themed picture books also significantly enhanced children’s empathy. Building on this finding, we further investigated empathy’s mediating role.

### Mediation analysis

**Figure 3:**
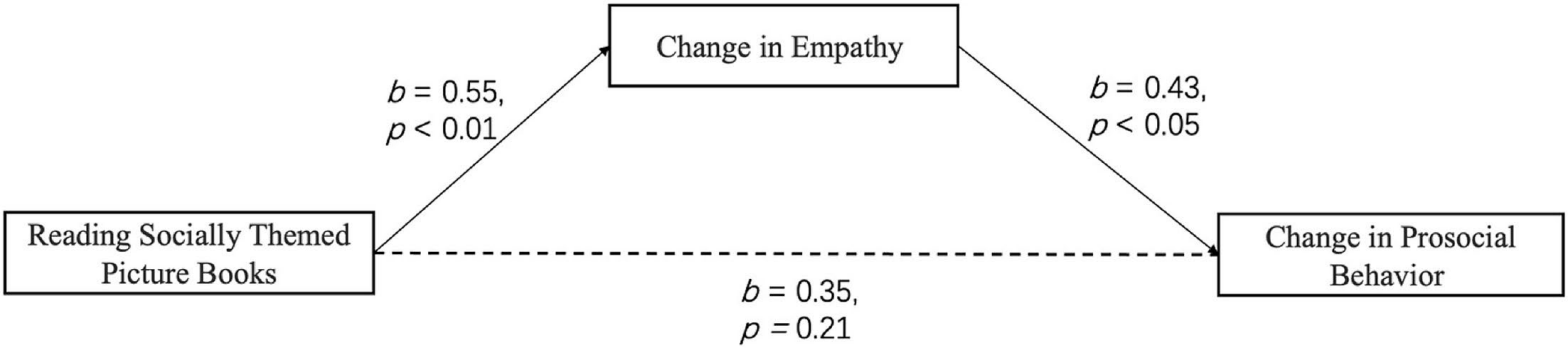
Complete mediated effect of empathy between intervention and prosocial behavior.

We used PROCESS 3.4.1 Model 4 to examine whether empathy mediates the relationship between reading socially themed picture books and pro-social development. The independent variable was whether children read socially themed picture books (social themes = 1; other themes = 0). We tested the mediation effect with the change in empathy (pre- and post-test difference) as the mediating variable, and the change in pro-social behavior (pre- and post-test difference) as the dependent variable. The results showed that reading socially themed picture books significantly predicted changes in children’s empathy (*b* = 0.55, *t* = 2.90, *df* = 56, *p* < 0.01). Additionally, empathy significantly predicted changes in pro-social behavior (*b* = 0.43, *t* = 2.33, *df* = 55, *p* < 0.05). However, the direct effect of reading socially themed picture books on changes in pro-social behavior was not significant (*b* = 0.35, *t* = 0.83, *df* = 55, *p* = 0.21) (see Figure 3). Mediation analysis confirmed that the change in empathy fully mediated the relationship between reading socially themed picture books and the change in pro-social behavior (see Table 4).

**Table 4:** Standardized mediation effects and 95% confidence intervals.

	*β*	*SE*	95% *CI*
Total effect	0.59	0.27	[0.04, 1.14]
Direct effect	0.35	0.28	[− 0.21, 0.92]
Indirect effect	0.45	0.14	[0.11, 0.56]

## Discussion

Previous research has affirmed that reading socially themed picture books enhances the social cognitive skills of children. Building on these empirical findings, we designed an intervention to enhance the prosocial behavior of children. Additionally, we explored the underlying mechanism of this effect, i.e., whether empathy serves as a mediating factor. We found that parent–child shared reading of picture books with social themes significantly promoted prosocial behavior in children, with empathy playing a complete mediating role.

The current findings underscore the unique contributions of socially themed picture books in promoting prosocial behavior. Storybooks convey psychologically externalized concepts that reflect the ideas, images, understandings, and values prevalent in different social groups. When children engage with these storybooks, they adopt and internalize their ideas and values (Ding et al., 2021). In the present research, the socially themed picture books covered themes of sharing, cooperating, helping, and making friends. From the perspective of Social Learning Theory (Bandura and Walters, 1977), when role models receive positive outcomes for engaging in prosocial behavior or face negative consequences for not doing so, children gain valuable opportunities for observational learning, which in turn enhances their prosocial behavior and guides their social interactions. Besides the content of the picture books, the format of shared reading may also have contributed to the preschoolers’ prosocial behavior, although it was not the independent variable in this study. Qualitative research has also shown that shared reading can be used to convey socioemotional information, with adults using stories to introduce children to prosocial themes and ethical values (Bhavnagri and Samuels, 1996). During shared reading, adults’ reading strategies, such as relating the text or illustrations to children’s personal experiences, asking questions, and encouraging dialogue (Vezzoli et al., 2020), further enhance children’s ability to comprehend social norms.

Future long-term follow-up studies can be conducted to assess whether parent–child shared reading of socially themed picture books has a sustained impact on the development of prosocial behavior in children. Additionally, with the advent of digital books and technologies like augmented reality, research can further explore the effectiveness of new technologies in promoting children’s prosocial behavior compared with traditional practices. Finally, we suggest that future interventions prioritize scaffolding shared reading by supporting parents in how to effectively communicate with their children, e.g., using open-ended questions, providing feedback, and being attuned to their child’s evolving abilities (Whitehurst et al., 1988), to achieve optimal intervention outcomes.

We found that empathy plays a mediating role in reading socially themed picture books to promote prosocial behavior. Socially themed picture books often depict interpersonal scenarios that highlight characters’ emotions and internal states (Ding et al., 2021), illustrating the causal relationships between these emotions and subsequent behaviors. For instance, in a picture book focused on helping, a character may show sadness when in need of help and happiness when being helped. Previous research has confirmed that parental discussions about emotions, explaining the antecedents and consequences of emotions, promote young children’s understanding of emotions and internal states, sensitivity to the feelings of others, and awareness of how and when to act on this understanding in a prosocial manner. For instance, mothers’ explanations of emotions have been associated with toddlers’ attempts to understand the emotional state of a distressed person (Garner, 2003) and positively predict children’s prosocial behavior during play (Garner et al., 2008). In contrast, mothers’ emotional comments during reading are negatively associated with children’s physical aggression (Garner et al., 2008). A similar study has confirmed that discussing internal states and prosocial behaviors with children after teachers read prosocial stories to preschoolers significantly increases preschoolers’ empathy and helping and sharing behaviors, whereas children who discuss concrete actions and physical states after reading or do not participate in discussions do not show significant increases in empathy or prosocial behaviors (Brazzelli et al., 2021).

This mechanism can be further explained by the Empathy-Altruism Hypothesis (Dovidio, 1991). This hypothesis suggests that feelings, emotions and empathy drive altruistic motivation. Emotional arousal activates the perception of others’ predicament, which in turn leads to altruistic motivation and promotes prosocial behavior (Persson and Kajonius, 2016). During parent–child shared reading of socially themed picture books, children perceive the emotions of story characters and, with parental guidance, get a deeper understanding of the causes and consequences of these emotions. This process enhances their empathy. When children empathize with story characters, they are more likely to transfer this feeling to real-life situations, become more sensitive to others’ needs, and engage in more helping and sharing behaviors.

The current research has several limitations that should be acknowledged. First, the sample may not be fully representative, as it mainly focused on urban kindergarten children, with 86.6% of their parents holding a bachelor’s degree or higher. Caregiver education has been found to be positively associated with positive emotion words, emotion questions, and emotion explanations (Tao et al., 2013). Thus, whether the promotion of prosocial behavior by reading socially themed picture books with parents can be generalized to children of low socioeconomic status must be further investigated. Second, the intervention period was limited to 8 weeks, which, while sufficient for observing short-term effects, may be inadequate for evaluating the long-term impacts of parent–child picture book reading. Moreover, our study design lacked a follow-up phase to test the maintenance of the intervention effects. Finally, although we hypothesized that socially themed picture books would provide parent–child dyads with more opportunities to discuss about emotions and their antecedents and consequences, we did not code parent–child interactions during storytelling in general and conversations about emotions in particular. Future research should explore whether the effects of socially themed picture books on young children’s socioemotional development are mediated by emotional conversations.

In conclusion, we validated the effectiveness of the intervention aimed at developing prosocial behavior in children through parent–child shared reading of socially themed picture books and explored its underlying mechanisms. Our work provides a low-cost, convenient, and effective intervention that does not require specific space or professional training while improving children’s empathy and prosocial behavior simply through the daily activity of parent–child storytelling. The current findings also offer new insights into the role of picture books reading in the social development of children and provide empirical evidence to support the enhancement of family education practices.

## Data Availability

The original contributions presented in the study are included in the article/supplementary material, further inquiries can be directed to the corresponding author.
